# Phosphate and Borate-Based Composite Interface of Single-Crystal LiNi_0.8_Co_0.1_Mn_0.1_O_2_ Enables Excellent Electrochemical Stability at High Operation Voltage

**DOI:** 10.3390/ma16103613

**Published:** 2023-05-09

**Authors:** Fu Long, Yuyang Liu, Guobin Zhu, Yan Wang, Honghe Zheng

**Affiliations:** College of Energy, Soochow University, Suzhou 215006, China

**Keywords:** lithium-ion batteries, cathode, LiNi_0.8_Co_0.1_Mn_0.1_O_2_, boric acid

## Abstract

The application of nickel-rich cathodes in lithium-ion batteries has been hampered by its rapid capacity/voltage fading and limited performance of rate. In this work, a passivation technique is used to create a stable composite interface on single-crystal LiNi_0.8_Co_0.1_Mn_0.1_O_2_ (NCM811) surface, which greatly improves the cycle life-span and high-voltage constancy of cathode with 4.5 and 4.6 V cut-off voltage. The improved Li^+^ conductivity of the interface enables a firm cathode–electrolyte interphase (CEI), which reduces interfacial side reactions, lowers the risk of safety hazards, and improves irreversible phase transitions. As a result, the electrochemical performance of single-crystal Ni-rich cathode are remarkably enhanced. The specific capacity of 152 mAh g^−1^ can be delivered at a charging/discharging rate of 5 C under 4.5 V cut-off voltage, much higher than 115 mAh g^−1^ of the pristine NCM811. After 200 cycles at 1 C, the composite interface modified NCM811 demonstrates outstanding capacity retention of 85.4% and 83.8% at 4.5 V and 4.6 V cut-off voltage, respectively.

## 1. Introduction

In recent years, nickel-rich layered oxide NCM811 has been intensively investigated as a cathode material for large-scale applications in electric vehicles because of its unique strengths in terms of theoretical specific capacity, operating potential, and fabrication cost [1,2,3,4]. Generally, much interest has been generated in the secondary spheres structure of polycrystalline NCM811 cathode due to its high-level first Coulombic efficiency for de-embedded Li^+^ storage. However, during battery operation, polycrystalline particles are susceptible to cracking due to inherent anisotropic volume changes, which accelerates the bulk structure degradation and aggravates interface side reactions, thus shortening the battery life [5,6,7].

Compared with the polycrystalline aggregates, the single-crystal NCM811 materials with micro-sized particles exhibit excellent electrochemical performance because of the integrated bulk structure and fewer grain boundaries, reducing the interface reaction caused by intergranular crack [8,9,10,11]. However, there are certain downsides to single-crystalline cathodes. Firstly, because of the smaller primary particles, the single-crystal NCM811 material has higher irreversible lithium consumption due to their longer Li^+^ diffusion path [12,13,14]. Additionally, surface residual lithium compounds (RLCs) emerging from the manufacturing and storage processes is a major and unavoidable fundamental flaw. Specifically, LiOH not only causes cathode slurry gelation, but it also combines with LiPF_6_ in the electrolyte to generate HF, which corrodes the electrode and causes transition metal ion dissolution. The Li_2_CO_3_ is also a detrimental substance since it decomposes when charged to 4.2 V, causing the battery to bulge and shortening its life [15,16,17,18]. Additionally, cationic mixing is caused by the low-valence metal ions moving to the Li^+^ layer and occupying the location of Li^+^. Since a similar ionic radius exists between the Li^+^ (0.76 Å) and Ni^2+^ (0.69 Å), the Ni^2+^ are prone to occupying the position of Li^+^ resulting in a barrier against subsequent Li^+^ transmission and making the irreversible Li^+^ consumption increase, accompanying with transition of crystal structure from layered over spinel to NiO-like rock salt phase [19,20,21]. What is worse, apart from the synthesis process, the Li/Ni mixing are closely related to the severe operating conditions [22,23,24] (such as extreme charging voltage, higher working temperature). Oxygen evolution occurs in all nickel-rich cathodes, and the potential of 4.2 V (vs. Li/Li^+^ for NCM811) is the onset of oxygen evolution, which is intimately related with the onset of H2-H3 phase transition and drastically increased as the cut off potential and temperature rising. The unfavorable triple phase transition is caused by the Li/Ni mixing and oxygen evolution reaction, which results in oxygen loss from the cathode materials and thus poses a safety problem. Even worse, this irreversible phase transition is aggravated when elevating the cut-off potential of cathodes and operating temperature [25,26,27].

For the interface and structure problem of high-nickel materials, some artificial particular protective coating material, such as metal oxides [28,29], phosphates [30,31], fluorides [32,33], borates [34,35], or lithium-containing binary metal oxides [36,37], are regarded as one of the most feasible strategies to combat these issues. Among the aforementioned substances, phosphate coatings exhibit advantages not only due to their brilliant structure stability which is caused by the strong bond of P-O but also for the special chemical activity. More specifically, Li_3_PO_4_ coating possesses high ionic conductivity [38], LiH_2_PO_4_ coating can react with Li_2_CO_3_, LiOH and the surface lithium impurities, AlPO_4_ coating appears to have superior thermal stability and constructs a robust interfacial layer, isolating the erosion of organic electrolyte to the matrix particles [39,40]. For example, Zhang et al. [40] propose an advanced surface improvement strategy for NCM811 with fabricating the Li_3_PO_4_ nano layer, which substantially enhances the electrochemical performance of NCM811 due to the derived fast Li^+^ diffusion CEI. As surveyed by Hu et al. [41], the coated substance of LiH_2_PO_4_ on the NCM811 surface can react with Li_2_CO_3_ and LiOH to form a homogenous passivation layer of Li_4_P_2_O_7_ with an average of about 35 nm in thickness while sintering, which effectively alleviate the side reaction. Liu et al. [29] introduced NaH_2_PO_2_ to construct an ultrathin and stable P-doped functional passivation layer, which not only enhances the stability of the cathode/electrolyte interface, but also inhibits the irreversible phase transformation and the generation of intergranular cracking. Cho et al. [38] coated AlPO_4_ on NCM811 via a wet-coating process to attain a decorated nano layer with 10 nm thickness. As a result, due to the highly covalent bonding property between Al and (PO4)^3−^, the rate performance and capacity retention are substantially improved, which provides the coating with resistance to electrolyte reactions. Since these substances can provide stable interfacial passivation effects, it is necessary to study their synergistic effect, especially at high cut-off voltage. In other uses than the coating strategy as mentioned above, electrolyte additives play a crucial role for Ni-rich cathode in passivating the NCM811 cathode and sustaining its long-term durability, especially in addressing the extremely unstable interface under high cut-off voltage for Ni-rich cathodes. Boron-containing compounds, due to the electron-deficient B atom, are prone to interacting with nucleophilic species (such as F^−^) and tend to form stale CEI due to the intense B-O bond, which is often utilized as electrolyte additives, especially at higher cut-off voltage conditions. For instance, Zhao et al. [42] reported that the superior electrochemical performance of the NCM811 cathode is capable of reaching a voltage of 4.6 V by utilizing a unique electrolyte system, which contains fluorinated carboxylate with a lithium difluoro (oxalate) borate and fluorinated carbonate additive. The stable inorganic electrode/electrolyte interface rich in B^−^ and F^−^ at high cut-off voltages prevents the collapse of the cathode structure and the continuous dissolution of transition metal ions as well as reducing electrochemical polarization. Liu et al. [43] employed a high-voltage bifunctional nitrile–borate molecule, tris(2-cyanoethyl) borate, as a film-forming additive for LiNi_0.88_Co_0.09_Mn_0.03_O_2_ cathode. The B-rich additives-derived CEI layer not only protects the cathode from degradation by interfacial side reactions at 4.7 V, but also enlarges the galvanic oxidation window of the electrolyte. Interestingly, application of boric acid on NCM811 surface not only removes residual lithium, but also forms a stable interfacial layer containing B-O compounds [35].

Furthermore, we compare three representative modification studies of NCM811. Sim et al. [6] constructed carbon coatings using super P carbon black to enhance the Li^+^ and electron transport capacity of the material. The modified cathode exhibited a capacity retention of 87.8% after 80 cycles at 0.5 C, while showing excellent rate performance. Zhang et al. [7] used a multi-step calcination method to dope fluorine in the NCM811 lattice. This controlled doping technique increased the Co^3+^ and lattice oxygen content, improving ion diffusivity and lattice stability. The doped cathode exhibited 86.6% capacity retention after 1 C and 100 cycles. Wang et al. [8] utilized polysiloxane to eliminate residual H_2_O on the NCM surface and inhibit the corrosion reaction of HF. After 120 cycles, the capacity retention of the polysiloxane-coated NCM811 reached 91.5% at 1 C.

Considering the superiorly favorable properties of phosphate and boron-containing materials for high nickel materials, in this work, we designed a multifunctional composite passivation interface with aluminum triphosphate (ALP) and boric acid (BA) from the perspective of clearing the surface residual lithium and improving the high cut-off voltage performance of the single-crystal NCM811. ALP is a widely used environmental protection coating, which demonstrates the advantages of good thermal stability and corrosion resistance, and BA has weak acidity, which is usually used to prepare heat-resistant and corrosion-resistant glass products. By constructing a phosphorus–boron functional interface layer on the surface of a high-nickel material, the surface residual lithium is reduced, and the capacity retention of the modified cathode has a significant improvement, especially at high voltage (4.5 V/4.6 V). In addition, high temperature and high-voltage performance, the performance of rate, and thermal stability of the modified electrode are significantly enhanced. Combined with common advantages of interfacial phosphorylation and interfacial borylation, this artificial functional passivation interface is projected to give significant benefits in terms of cycle stability and thermostability at high operating potentials, making single-crystal NCM811 an attractive cathode material.

## 2. Materials and Methods

### 2.1. Preparation of Materials

**Figure 1:**
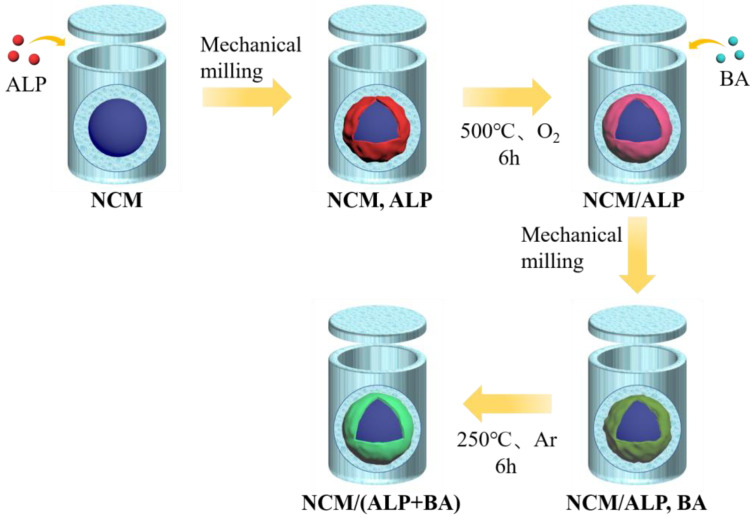
Synthesis diagram of modified single-crystal NCM811.

The experimental NCM811 powder was purchased from Dangsheng Ltd., Co., Beijing, China. The synthesis diagram of modified NCM811 as depicted in Figure 1, firstly, the precursor was obtained by mixing 20 mg ALP (AlH_2_P_3_O_10_, AR, Shanghai Aladdin Biochemical Technology Co., Ltd., Shanghai, China) with 2g NCM811 by mechanical milling and then the obtained mixing powder was calcinated at 500 °C with an O_2_ atmosphere for 6 h in a tube furnace. Secondly, 10 mg and 20 mg BA (H_3_BO_3_, GR, Shanghai Aladdin Biochemical Technology Co., Ltd., Shanghai, China) were added to the precursor for further mechanical milling and then calcined in an Ar atmosphere with 6 h at 250 °C to get the final products with different BA mass ratios. Accordingly, the bare single-crystal NCM811 and the two treated final samples were designated as SCNCM, SCNCM/(ALP+BA)-1 (10 mg BA), and SCNCM/(ALP+BA)-2 (20 mg BA), respectively. 

### 2.2. Preparation and Testing of Electrodes

The anode powder, polyvinylidene fluoride, and conductive carbon black (weight ratio 88:5:7) was fully mixed in N-methyl pyrrolidone solvent. The cathode slurry was thoroughly mixed under Ar atmosphere and coated in aluminum foil (12 mm thick). The cathode pole piece was calendered and cut into slices (13 mm diameter) after drying at 60 °C for 6 h. The original and modified samples have similar mass loadings of 4.0 ± 0.1 mg cm^−2^ and the prepared cathode pole piece was dried for 16 h at 120 °C under vacuum. The rough loading of graphite anode materials and NCM811 cathode was around 2.7 and 4.6 mg cm^−2^ for a full cell.

The electrochemical properties of the cathodes were tested in CR2032 coin-type cells. The adopted separator between the cathode and the anode was a porous polypropylene film (Celgard 2500) while lithium foil was used as the counter electrode. The electrolyte was obtained from Capchem Ltd., Co. China, and is prepared by dissolving 1 mol L^−1^ LiPF_6_ in a ternary solvent of ethylene carbonate (EC), ethyl methyl carbonate (EMC), and dimethyl carbonate (DMC) at a weight ratio of 1:1:1. Three formation cycles were applied between 2.8 and 4.5 and 4.6 V vs. Li/Li+ at a 0.1 C rate (1 C = 200 mA g^−1^) on a Maccor S4000 battery cycler (Maccor Instruments, USA) at 25 °C (or 55 °C).The rate performance of the electrodes was tested with different charge/discharge rates (0.1, 0.2, 0.5, 1, 2, and 5 C, respectively). Long-term cycling capability was compared with 1 C charge and discharge rates. Electrochemical impedance spectroscopy (EIS) information was collected in the frequency range of 10^5^ to 10^−2^ Hz with an amplitude of 5 mV on an IM6 electrochemical workstation (Zahner, Germany) at 25 °C at a certain electrode potential. The thermal stability of charged cathodes was evaluated by differential scanning calorimeter. The cathodes were first charged to 4.5 V at 0.1 C and then held at 4.5 V for an additional 1 h. The cathode materials were collected from the dismantled cells and then washed with DMC to remove remaining electrolytes and dried. Charged cathode powders (5.0 mg) were mixed with 3 μL of electrolyte in a hermetic aluminum pan. The temperature was raised from ambient to 400 °C at a continuous heating rate of 10 °C min^−1^ during the differential scanning calorimetry experiments. The relevant parameters of material characterization are included in the supporting information.

### 2.3. Material Characterization

The crystalline phase of the NCM811 material was determined by using X-ray diffraction (XRD, Rint-2000, Rigaku with 0.71 nm CuKα) at a scan rate of 3° min^−1^ between 10° and 80°. Morphologies of the samples were observed by scan electron microscope (SEM, Hitachi S-8010, 10 kV). Element distribution was examined by using an energy dispersive X-ray (EDX) spectrometer coupled to Hitachi S-8010 equipment. Transmission electron microscopy (TEM, FEI Tecnai G-20) micrography were captured at an acceleration voltage of 200 kV. X-ray photo-electron spectroscopy characterization (XPS) was conducted on a VG Multilab 2000 apparatus (Al:1486.7 eV). The NCM811 electrodes after cycling were obtained from dismantled batteries and cleaned with DMC solvent and then dried under vacuum at 60 °C for 2 h.

## 3. Results and Discussion

**Figure 2:**
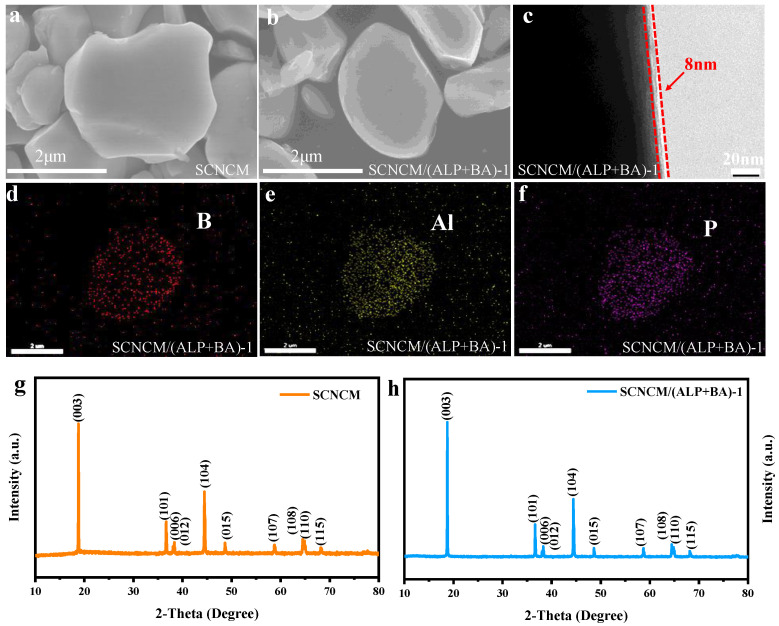
(**a**) SEM image of SCNCM sample, (**b**,**c**) SEM and TEM images of modified samples, (**d**–**f**) the distribution of B, Al, and P elements for SCNCM/(ALP+BA)-1 powder sample, (**g**–**h**) XRD patterns of SCNCM and SCNCM/(ALP+BA)-1 sample.

The SEM images of the original SCNCM and SCNCM/(ALP+BA)-1 samples are shown in Figure 2a,b. The diameter of the original SCNCM particles is about 2–3 µm and it has a smooth and clean surface. However, homogeneous coating can be clearly observed in SEM and TEM images (Figure 2c) of SCNCM/(ALP+BA)-1 sample, suggesting a composite interfacial layer has been formed by bonding reaction with residual lithium on the surface of NCM811 particles. In addition, the spatial elements distribution of SCNCM/(ALP+BA)-1 sample was obtained by the SEM-Energy dispersive X-ray spectroscopy (EDS) and the elements (B, Al, and P) were equally distributed over the SCNCM/(ALP+BA)-1 sample (Figure 2d–f). This indicates that the ultrathin uniform passivation layer is produced successfully on the SCNCM surface. Moreover, the crystallographic structures of sample were determined by XRD tests and presented in Figure 2g,h. All XRD patterns correspond to a well-defined α-NaFeO2 rhombohedral structure (space group of R-3m) [44]. The splitting of (108)/(110) and (006)/(102) peaks in the SCNCM/(ALP+BA)-1 verifies the ordered layered structure and indicates that the fundamental structure of the SCNCM is not damaged during the modified process. The degree of Li/Ni cation mixing can be reflected by calculating the intensity ratio of the peak (003) to peak (104), and a higher value of I (003)/I (104) implies a more sequential layer structure. The value of I (003)/I (104) is 2.01 for the SCNCM sample and 2.41 for the SCNCM/(ALP+BA)-1 sample, signifying the Li/Ni mixing in the SCNCM/(ALP+BA)-1 sample is beneficially reduced [20]. This may be attributed to the surface reconstruction during chemical conversion and Al^3+^ enter into the Ni site under high temperature re-treatment process. For the pristine SCNCM, SCNCM/(ALP+BA)-1, and SCNCM/(ALP+BA)-2, the BET surface area is obtained to be 1.42, 1.39, and 1.44 m^2^ g^−1^, respectively.

**Figure 3:**
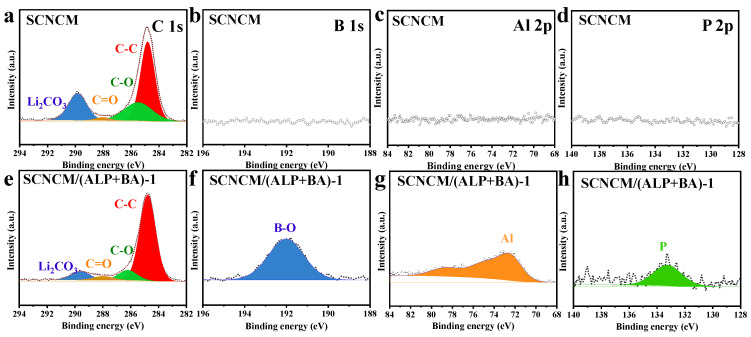
(**a**–**d**) XPS fitting results of C 1s, B 1s, Al 2p, and P 2p of SCNCM and (**e**–**h**) of SCNCM/(ALP+BA)-1.

The surface element sensitive characterization method XPS was used to evaluate the samples in order to furthermore investigate the compound composition of the coating. The XPS findings of the SCNCM and SCNCM/(ALP+BA)-1 samples are shown in Figure 3, and the accuracy of the calibration spectrum depends on a C1s peak of 284.8 eV. As shown in Figure 3a,e, the C1s peak with reduced intensity demonstrates that the interfacial reactions between the ALP/B and RLCs since the Li_2_CO_3_ content originating from the surface residual lithium compounds is decreased. In addition, the characteristic B, Al, and P peaks are only detected on the SCNCM/(ALP+BA)-1 sample as expected. Specifically, as shown in Figure 3f–h, binding energy at 191.8 eV for the B-O bond originated from the borates and the binding energy of 133.3 eV represents a metal phosphate, which indicates the formation of a phosphorus boronated bi-functional passivation layer with AlPO_4_, Li_3_PO_4_ and lithium boron oxide (Li_3_BO_3_, B_2_O_3_).

**Figure 4:**
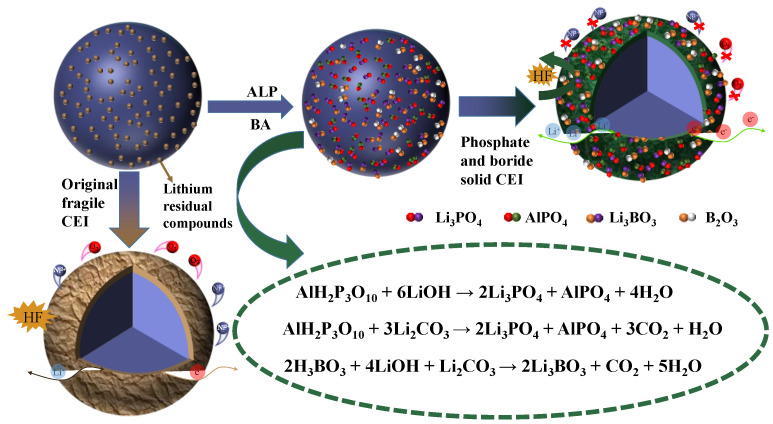
Schematic diagram for the interface reaction principle of ALP and BA on NCM811 sample.

The schematic diagram of the construction process of the functional passivation coating on the single-crystal NCM811 surface appears in Figure 4. The function-specific ALP and BA substances not only possess the chemical activity to react with the residual lithium compounds (LiOH and Li_2_CO_3_), but the resulting by-products are also conducive to the construction of stable CEI, which can be an efficient solution to the interfacial problem of the NCM811 cathode. On the one hand, the stable target product of AlPO_4_ and B_2_O_3_ can serve as a solid barrier to safeguard the matrix particles from harmful substances produced by electrolyte decomposition at higher operation voltage (4.5 V/4.6 V) and the boron-containing compounds can interact with the HF resulting from the decomposition of lithium salt in the electrolyte to form B-F compounds due to the fluorophilic property of boron, which significantly reduces degradation of the active material by HF, inhibits the dissolution of transition metals, alleviates the side reactions in interface areas, delays the serious structural degradation of high-nickel materials, and prolongs the life of a cell under high-voltage working conditions. On the other hand, not only the crystalline Li_3_PO_4_ can be selected as a channel to facilitate the transfer of Li^+^ but the Li_3_BO_3_ with high ionic conductivity can also effectively promote the diffusion of Li^+^, resulting in a low impedance phosphate–boron interface layer being formed. It is because of the synergy of the two functional species that the battery exhibits an excellent electrochemical property.

**Figure 5:**
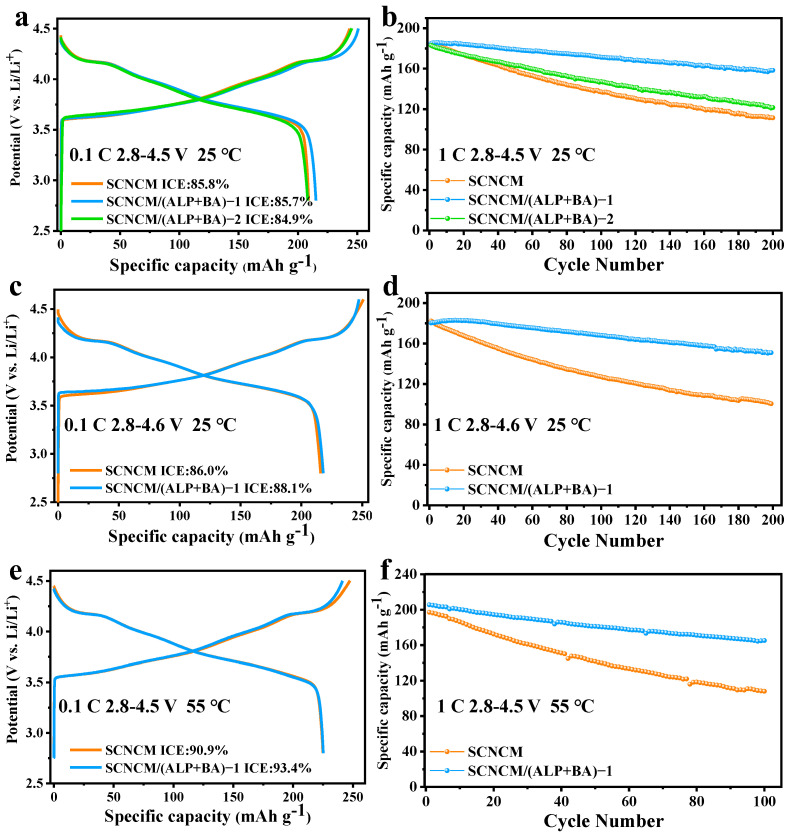
The first charging and discharging curves of the SCNCM, SCNCM/(ALP+BA)-1, and SCNCM/(ALP+BA)-2 samples at the current of 0.1 C with the voltage range of (**a**) 2.8−4.5 V at 25 °C and (**c**) 2.8−4.6 V at 25 °C, and (**e**) 2.8−4.5 V at 55 °C, the cycling graph of the SCNCM, SCNCM/(ALP+BA)-1, and SCNCM/(ALP+BA)-2 samples at the current of 1 C in the voltage range of (**b**) 2.8−4.5 V at 25 °C, (**d**) 2.8−4.6 V at 25 °C, and (**f**) 2.8−4.5 V at 55 °C.

We tested the electrochemical performance of various electrode materials under error to verify the positive effect of the composite grafted ALP/BA layer on NCM811. The charge/discharge patterns in the first cycle between 2.8 and 4.5 V at 0.1 C and 25 °C are displayed in Figure 5a. The first charge/discharge-specific capacities of the different electrodes are 244/209, 251/215, and 245/208 mAh g^−1^ and the calculated initial Coulombic efficiency (ICE) is 85.8, 85.7, and 84.9%, respectively. Compared with the pristine cathode, the modified sample of SCNCM/(ALP+BA)-1 delivers a higher specific capacity while maintaining a similar ICE, which indicates a more optimal Li^+^ deintercalation–intercalation process. However, for the SCNCM/(ALP+BA)-2 sample, it exhibits relatively low ICE and discharge specific capacity, indicating that this is not the optimal modification conditions, which should be caused by an excess of boric acid. Furthermore, Figure 5b characterizes the cycling stability of all electrodes at room temperature over a long period of time. After 200 cycles under 1C, the modified electrode of SCNCM/(ALP+BA)-1 has a reversible specific capacity of 158 mAh g^−1^ and a capacity retention up to 85.4%. The reversible specific capacity of the SCNCM/(ALP+BA)-2 was 122 mAh g^−1^ with a capacity retention of 66.4%, whereas the matching results for the bare SCNCM electrode are significantly lower (111 mAh g^−1^ and 60.2%), denoting the cycling stability of the modified SCNCM/(ALP+BA)-1 electrode has been dramatically improved. To emphasize the remarkable performance of ALP/BA layer, we further increased the cut-off voltage of the SCNCM/(ALP+BA)-1 electrode to 4.6 V and also tested the higher temperature and higher voltage performance (4.5 V, 55 °C). The electrochemical performance of the SCNCM/(ALP+BA)-1 electrode with 4.6 V is presented in Figure 5c,d. The first charge/discharge specific capacities of the SCNCM and SCNCM/(ALP+BA)-1 electrodes were 251/216 and 247/218 mAh g^−1^, the corresponding ICE was 86.0% and 88.1%, respectively. In addition to the enhanced ICE, the capacity retention rate of the SCNCM/(ALP+BA)-1 electrode (83.8%) is much greater than that of the primitive electrode (55.1%). It is easy to find that as the cut-off voltage rises, the decaying trend of the cycling performance for the modified electrode is slower than a reference electrode, which indicates that this strategy of using interfacial reactions to generate a composite passivation layer has superior effects, especially the formation of this boron-rich and aluminium-rich CEI greatly extends the life of the cell at high operating voltages. As illustrated in Figure 5e,f, the first charge/discharge specific capacities of SCNCM electrode and SCNCM/(ALP+BA)-1 electrode were 247/225 and 241/225 at 4.5 V cut-off voltage and 55 °C, respectively, and the correlation capacity retention rates were 54.8% and 80.3% after 100 cycles. Such intensification of thermal stability can be associated with the formation of robust CEI, which effectively protects the electrode material from electrolyte attack and mitigates bulk structural degradation of the cathode material under harsh operating conditions [45,46].

**Figure 6:**
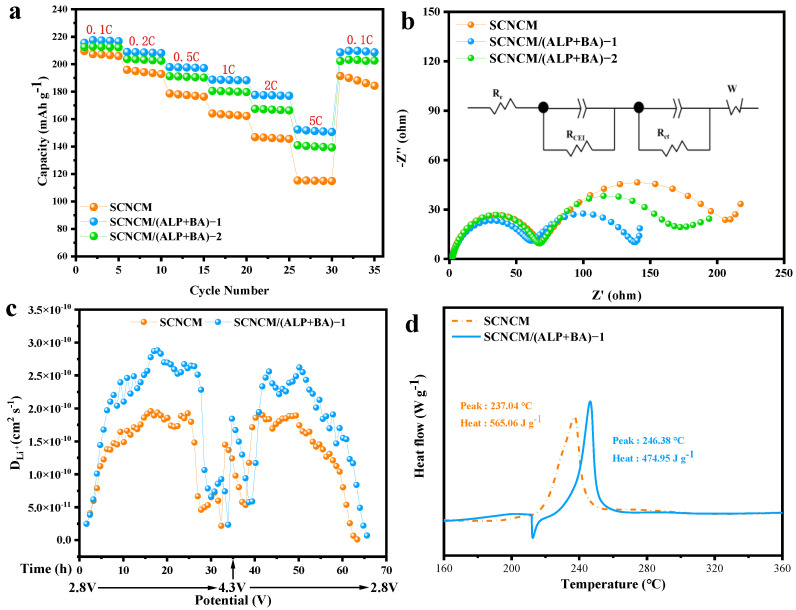
(**a**) Rate property of the different electrodes, (**b**) Nyquist plots of different electrode after 200 cycles with 2.8–4.5 V at 1C, (**c**) the calculated diffusion coefficients of SCNCM and SCNCM/(ALP+BA)-1 electrodes from GITT tests after a complete charge/discharge process, (**d**) differential scanning calorimetry (DSC) measurement of pristine SCNCM and SCNCM/(ALP+BA)-1 charged to 4.5 V vs. Li/Li^+^.

Figure 6a depicts the rate performance of these electrodes. Clearly, the SCNCM/(ALP+BA)-1 electrode revealed the best performance of rate and maintained a specific capacity of 152 mAh g^−1^ at 5 °C, much higher than that of 115 mAh g^−1^ of the SCNCM cathode and 139 mAh g^−1^ for the SCNCM/(ALP+BA)-2 electrode. Moreover, when the circulation rate retreats to 0.1 C, as compared with the SCNCM electrode (191 mAh g^−1^), the SCNCM/(ALP+BA)-1 electrode exerts a higher specific capacity (209 mAh g^−1^) and the recovered specific capacity tends to be more stable. Under the harsh conditions of 4.5 V cut-off voltage, the ALP/BA decorated NCM811 performs outstandingly both in the long-term cycling and capacity retention at high circulation rate [47,48]. Such great enhancement of electrochemical properties implies that the SCNCM/(ALP+BA)-1 sample exhibits an acceleration of the electrode reactivity, which is attributed to the CEI formed by the participation of ALP/BA. To explore the electrode kinetics, the Nyquist plots of the SCNCM and SCNCM/(ALP+BA)-1 cathodes after cycling 200 at 4.5 V are shown in Figure 6b. The Nyquist plots consist of the electrolyte resistance (R_e_), the cathode/electrolyte interphase impedance (R_CEI_) in the high-frequency region corresponds to the Li^+^ migration through the surface insulating layer of active material particles, the charge-transfer resistance (R_CT_) in the medium-frequency region reflects the charge transfer between electrons and ions at the conductive junction, and the Warburg impedance in the low-frequency region represents the solid diffusion process of Li^+^ in the active material particles. Remarkably, the values of R_CEI_ and R_CT_ for the SCNCM/(ALP+BA)-1 electrode (58.5 and 79.4 Ω) are smaller than the reference SCNCM (64.4 and 146.6 Ω), especially for the R_CT_ value, which leads to the conclusion of a significant reduction in the charge transfer resistance on the particle surface. As a result, due to the involvement of the coating substance, a robust and lower impedance CEI is generated during electrochemical cycling. Furthermore, as shown in Figure S1, we came to a similar conclusion by analyzing the Nyquist plots of electrodes at a cut-off voltage of 4.6 V, compared with pristine electrode (319.4 Ω), the R_CT_ values for the treated electrode (219.4 Ω) was significantly lower. Furthermore, the galvanostatic intermittent titration technique (GITT) can more sufficiently explain the improvement in rate cycle stability, and the calculation results of the Li^+^ diffusion coefficient are shown in Figure 6c. The Li^+^ diffusion coefficient of the modified electrode is higher than the pristine electrode at 4.5 V cut-off voltages during a charge/discharge process, this indicates that the modified samples possess faster Li^+^ transport. The improvement in Li^+^ transport rate can be attributed to the surface conductive lithium salt, which is formed by the reaction of ALP and BA with residual lithium compounds. In order to evaluate the thermal performance of the cathode materials, DSC measurements were performed as the electrode charged to 4.5 V. As Figure 6d shows, the exothermic reaction of SCNCM/(ALP+BA)-1 occurred at a higher temperature of 246.38 °C with a heat generation of 474.95 J g^−1^, compared with the data of 237.04 °C and 565.06 J g^−1^ for the reference sample, respectively. The exothermic reaction is affected by the release of oxygen during the decomposition of the high-nickel cathode materials. These outstanding thermal behaviors of the modified cathode materials may be related to the phosphate compounds and borate compounds that are very stable against both the chemical and thermal reactions owing to the strong PO_4_^3−^ and BO_3_^3−^ bonding, which make the decrease of cathode surface exposed to the electrolyte to reduce the generation of oxygen due to a side interface reaction so that the SCNCM/(ALP+BA)-1 is able to reduce the amount of heat release and delay the thermal peak.

**Figure 7:**
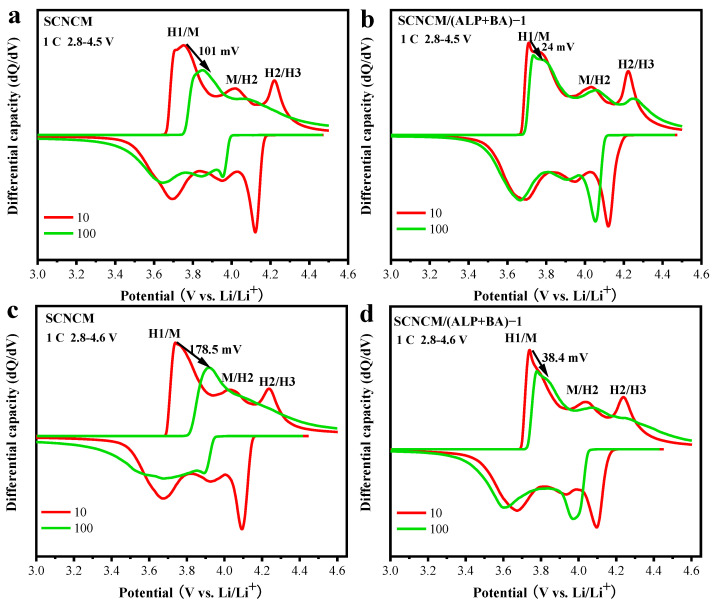
The dQ/dV curves (**a**,**c**) for SCNCM at 4.5 V and 4.6 V cut-off voltage and (**b**,**d**) for SCNCM/(ALP+BA)-1 electrode during charge/discharge at different electrochemical cycles.

The differential capacity versus electrode potential (dQ/dV) curves were calculated by differentiating the 10th and 100th charge–discharge curves to reveal the phase transition behavior during deep electrochemical cycles. By analyzing the differential capacity curves at different cut-off voltages (Figure 7a–d), three different redox peaks can be observed in the previous curves, representing the phase transition from H1 to M, from M to H2, and from H2 to H3, respectively. However, after 100 cycles, the peaks representing the H2-to-H3 phase transition have completely disappeared for the pristine electrode, and the peaks are clearly observed in the curves of the modified electrode, which suggests that this irreversible phase transition of the material from layered to spinel and deleterious rock salt structures is significantly suppressed during multiple electrochemical cycles. This irreversible phase transition behavior becomes more severe as the cut-off voltage is increased to 4.6 V. For the SCNCM electrode, the peaks representing the M-to-H2 and H2-to-H3 phase transitions have completely disappeared after 100 cycles, however, these two characteristic peaks are observed in the SCNCM electrode. Furthermore, the anodic peaks of the SCNCM electrode shift significantly to higher potential with increasing electrochemical cycles, which indicates a serious polarization of the SCNCM electrode. It is such a severe irreversible phase transition that causes a dramatic decrease in the plateau voltage and capacity [27]. By building a stable CEI, the ALP/BA passivation layer becomes a solid barrier between the surface of active material and the electrolyte, greatly alleviating the interfacial side reactions and protecting the structural stability of the cathode material. Eventually, the modified cathode performs superior electrochemical behavior.

**Figure 8:**
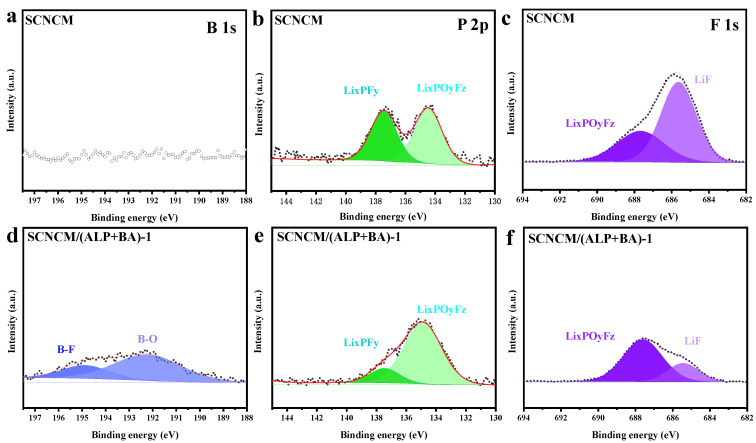
(**a**–**c**) XPS fitting results of B 1s, P 2p, and F 1s for SCNCM cathode after 200 cycles and (**d**–**f**) for SCNCM/(ALP+BA)-1 cathode.

To further explore the factors of capacity degradation, a comparative analysis of the electrode surfaces of SCNCM and SCNCM/(ALP+BA)-1 cathodes was analyzed relying on XPS. The XPS fitting results of the electrodes after 200 cycles at 4.5 V cut-off voltage in the region of C 1s, F 1s, and Ni 2p are displayed in Figure 8. As shown in Figure 8a,d, the B 1s signals are detected in the SCNCM/(ALP+BA)-1 sample and the B-O bond (192.4 eV) and B-F bond (194.9 eV) peaks appear in the B1s spectrum. A plausible explanation is that borates are participating in the formation of CEI during the electrochemical cycling, which forms a protective barrier against the corrosion of HF by forming B-F or B-O-based compounds. Accordingly, the presence of B-rich CEI on the surface of SCNCM/(ALP+BA)-1 inhibits the dissolution of transition metal ions, interfacial side reactions of the electrode/electrolyte and irreversible phase transition. Furthermore, P 2p spectra (Figure 8b,e) reveal a critical point, the relative intensity of the peak corresponding to Li_x_PF_y_ (137.5 eV) is stronger in SCNCM, indicating a more severe decomposition behavior of the lithium salt (LiPF6). Additionally, from Figure 8c,f, the F1s peak in the SCNCM/(ALP+BA)-1 sample shows mainly two components LiF (685.4 eV) and Li_x_PF_y_ (687.6 eV), both of which are produced by the decomposition of the lithium salt on the electrode surface. The significantly weaker intensity of the F1s peak of SCNCM/(ALP+BA)-1 compared to SCNCM indicates a milder side effect during CEI formation. In addition, the morphologies of SCNCM and SCNCM/(ALP+BA)-1 electrodes after cycling at 4.5 V cut-off voltage were shown in Figure S2. Due to the super structural stability of the single crystal, the single-crystal NCM811 still maintains the original particle-like structure after cycling, but the interface of these samples is completely different. The surface of the SCNCM particles is clearly covered with deposits that come from the products of severe electrolyte decomposition reactions, making the surface rough and uneven, and the SCNCM/(ALP+BA)-1 particles exhibit a uniform and smooth surface, further demonstrating that the multifunctional composite passivation interface has the function of mitigating electrolyte side reactions and constructing stable CEI.

We assembled full cells with graphite anode to achieve a better evaluation of this modification strategy in terms of applications. The first charge/discharge profiles, first between 2.8 and 4.3 V at 0.1 C and 25 °C, are shown in Figure S3. The charge/discharge specific capacity of the first circle for SCNCM-FC and SCNCM/(ALP+BA)-FC electrodes are 241/204 and 241/203 mAh g^−1^, and the corresponding ICE is 84.4% and 84.1%. Although the two electrodes have similar ICE, the long-term cycling performance differs significantly. As illustrated in Figure S3b, the full cell of SCNCM/(ALP+BA)-FC cathode achieved a capacity retention of 86.8% after 200 cycles at 1 C, while only exhibiting 79.3% capacity retention for the full cell of unmodified SCNCM cathodes. Experiments on full cells further demonstrate the feasibility of this modification strategy, even in the field of applied electrochemistry.

## 4. Conclusions

In this study, in order to strengthen the performance of single-crystal NCM811 cathode at high operation voltage, we designed a multifunctional composite passivation interface with ALP and BA from the perspective of clearing the surface residual lithium and enhancing the high-voltage performance of the single-crystal NCM811 cathode. Specifically, the stable by-products AlPO_4_ and B_2_O_3_ serve as a solid barrier to shield the substrate from the attack of hazardous substances created by electrolyte decomposition at high operation voltage, while the crystalline Li_3_PO_4_ and L_3_BO_3_ act as a selective channel facilitating the transfer of Li^+^. As a result, the cycling performance of the cathode is notably improved at 4.5 V and 4.6 V. Under 4.5 V cut-off voltage, a specific capacity of 152 mAh g^−1^ can be delivered at a charging/discharging rate of 5 C, while the original cathode is significantly lower exhibiting only 115 mAh g^−1^. Even at high cutoff voltages of 4.5 V and 4.6 V, the modified samples performed excellent capacity retention of 85.4% and 83.8% after 200 cycles at 1 C, respectively. In addition, the harsh high-voltage-temperature (4.5 V, 55 °C) performance of the modified cathode increased from 54.8% to 80.3% after 100 cycles and the improvement of heat resistance was realized. This functional passivation interface is projected to give significant benefits in both performance of rate and cycle stability at high operating potentials, making single-crystal NCM811 an attractive cathode material for high-energy LIB. 

## Data Availability

The datasets used and/or analyzed in the present study are available from the corresponding author upon reasonable request.

## Supplementary Materials

The following supporting information can be downloaded at: https://www.mdpi.com/article/10.3390/ma16103613/s1. Table S1. XRD data for SCNCM and SCNCM/(ALP+BA)-1. Table S2. Comparison table of NCM study results. Figure S1. (a) Nyquist plots of SCNCM and SCNCM/(ALP+BA)-1 electrode after 200 cycles at 1 C within 2.8–4.6 V, (b) the calculated diffusion coefficients of SCNCM and SCNCM/(ALP+BA)-1 electrodes from GITT tests during a complete charging and discharging process at 2.8–4.6 V. Figure S2. The SEM images of (a) SCNCM and (b) SCNCM/(ALP+BA)-1 cathodes after 200 cycles at 4.5 V. Figure S3. The first charge and discharge curves of the SCNCM-FC and SCNCM/(ALP+BA)-FC, at the current of 0.1 C in the voltage range of 2.8−4.3 V, (b) cycling performance of the SCNCM-FC and SCNCM/(ALP+BA)-FC electrode at the current of 1 C in the voltage range of 2.8−4.3 V. Refs. [49,50,51,52,53] were cited in the Supplementary Materials.
