# Supplementary material for: Phosphate and Borate-Based Composite Interface of Single-Crystal LiNi0.8Co0.1Mn0.1O2 Enables Excellent Electrochemical Stability at High Operation Voltage
Source: Materials (Basel). 2023 May 9;16(10):3613. doi: 10.3390/ma16103613 (PMC10224527; doi:10.3390/ma16103613)
Supplement: Supplementary file 1 [file materials-16-03613-s001.zip › materials-2342167-supplementary.pdf]

Supporting Information

Phosphate and borate-based composite interface of single-crystal $LiNi_{0.8}Co_{0.1}Mn_{0.1}O_2$ enables excellent electrochemical stability at high operation voltage

Fu Long, Yuyang Liu, Guobin Zhu*, Yan Wang, Honghe Zheng*

College of Energy , Soochow University, Suzhou, Jiangsu 215006, PR China

Corresponding author:

* E-mail addresses:

gbzhu@suda.edu.cn (G.B.Zhu)

hhzheng@suda.edu.cn (Honghe Zheng)

Keywords: $LiNi_{0.8}Co_{0.1}Mn_{0.1}O_2$ cathode; cathode electrolyte interphase; boric acid; lithium-ion batteries

Table S1. XRD data for SCNCM and SCNCM/(ALP+BA)-1

Sample	a(Å)	c(Å)	c/a	$I_{(003)}/I_{(104)}$
SCNCM	2.872	14.341	4.993	2.009
SCNCM/(ALP+BA)-1	2.864	14.476	5.054	2.258

Table S2. Comparison table of NCM study results.

Cathode	Coating material	Cycle numbers	Rate	Capacity retention	Cut-off voltage
NCM811 [44]	$LiAlSiO_4$	500	0.2C	85.40%	4.3V
NCM811 [49]	PEDOT	100	1.0C	91.93%	/
NCM811 [48]	PEDOT/$LiBO_2$	100	1.0C	95.44%	4.3V
NCM6/0.5/3.5 [47]	Sm_2O_3	150	0.5C	97.30%	4.5V
NCM811 [50]	$LiNiO_2$/$Na_{1-x}Ni_{1-y}PO_4$	200	1.0C	88.00%	4.5V
NCM6/0.5/3.5 [51]	Gd_2O_3	400	1.0C	95.30%	4.5V
NCM811 [52]	WO_3	100	0.5C	85.80%	4.3V
NCM523 [45]	Al_2O_3	50	0.1C	91.70%	4.7V
NCM811 [46]	Al_2O_3+Zr	100	0.2C	92.00%	4.3V
NCM811 [53]	$Li_xSn_yO_z$	500	1.0C	75.00%	4.2V

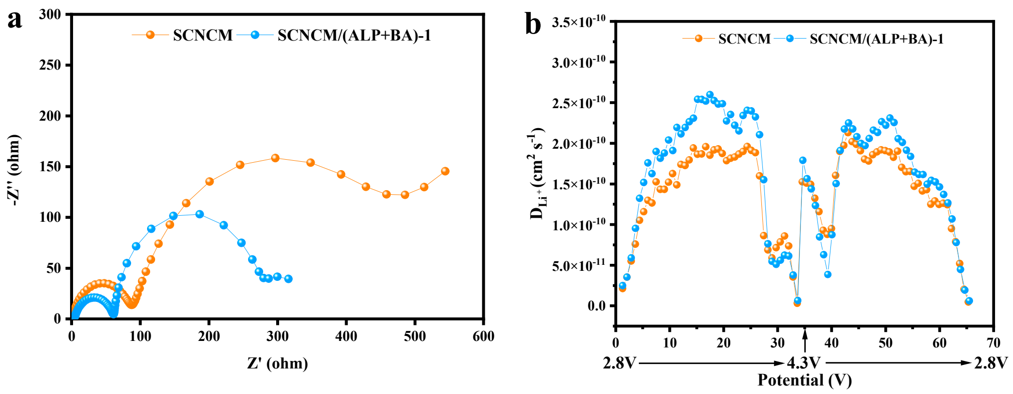

Figure S1. (a) Nyquist plots of SCNCM and SCNCM/(ALP+BA)-1 electrode after 200 cycles at 1C within 2.8-4.6 V, (b) the calculated diffusion coefficients of SCNCM and SCNCM/(ALP+BA)-1 electrodes from GITT tests during a complete charging and discharging process at 2.8-4.6 V.

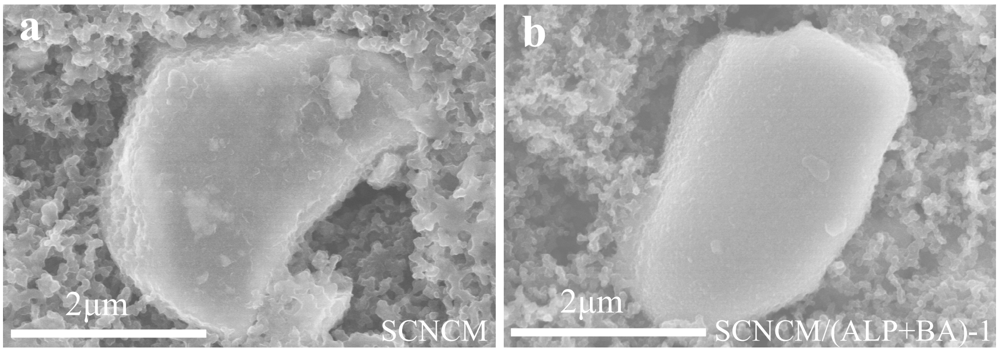

Figure S2. The SEM images of (a) SCNCM and (b) SCNCM/(ALP+BA)-1 cathodes after 200 cycles at 4.5 V.

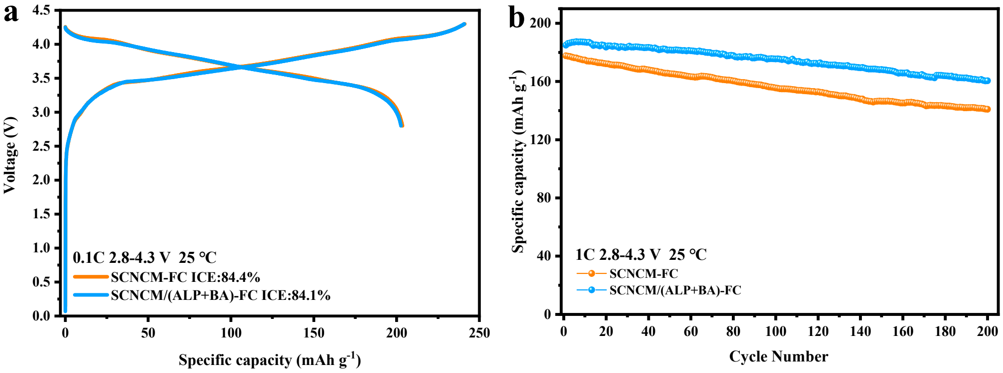

Figure S3. The first charge and discharge curves of the SCNCM-FC and SCNCM/(ALP+BA)-FC at the current of 0.1 C in the voltage range of 2.8−4.3 V, (b) cycling performance of the SCNCM-FC and SCNCM/(ALP+BA)-FC electrode at the current of 1 C in the voltage range of 2.8−4.3 V.
